# Efficacy of tranexamic acid in reducing allogeneic blood products in adolescent idiopathic scoliosis surgery

**DOI:** 10.1186/s12891-016-1006-y

**Published:** 2016-04-27

**Authors:** Wen-yuan Sui, Fang Ye, Jun-lin Yang

**Affiliations:** Department of Orthopaedics, The 1st Affiliated Hospital of Sun Yat-sen University, Zhongshan Er Road Yuexiu District, Guangzhou, China; Department of anesthesia, The 1st Affiliated Hospital of Sun Yat-sen University, Guangzhou, China

**Keywords:** Adolescent idiopathic scoliosis, Tranexamic acid, Blood transfusion, Blood loss

## Abstract

**Background:**

Adolescent idiopathic scoliosis (AIS) surgery usually require prolonged operative times with extensive soft tissue dissection and significant perioperative blood loss, and allogeneic blood products are frequently needed. Methods to reduce the requirement for transfusion would have a beneficial effect on these patients. Although many previous studies have revealed the efficacy of tranexamic acid (TXA) in spinal surgery, there is still a lack of agreement concerning the reduction of both blood loss and transfusion requirements of large dose tranexamic acid (TXA) in surgery for adolescent idiopathic scoliosis (AIS). The objective of this study was to elevate the efficacy and safety of a large dose tranexamic acid (TXA) in reducing transfusion requirements of allogeneic blood products in adolescent idiopathic scoliosis (AIS) surgery using a retrospective study designed with historical control group.

**Methods:**

One hundred thirty seven consecutive AIS patients who underwent surgery treatment with posterior spinal pedicle systems from August 2011 to March 2015 in our scoliosis center were retrospectively reviewed. Patients were divided into two groups, the TXA group and the historical recruited no TXA group (NTXA). Preoperative demographics, radiographic parameters, operative parameters, estimated blood loss (EBL), total irrigation fluid, number of patients requiring blood transfusion, mean drop of Hb (Pre-op Hb-Post-op Hb), haematocrit pre and post-surgery, mean volume of blood transfusion, hospitalization time, and adverse effect were recorded and compared.

**Results:**

All the patients were successfully treated with satisfied clinical and radiographic outcomes. There were 71 patients in the TXA group and 66 patients in the NTXA group. The preoperative demographics were homogeneity between two groups (*P* > 0.05). There were no significant difference in average operative time between two groups (209 min vs 215 min, *p* >0.05). Number of patients in the TXA group showed a significant decrease in transfusion requirements with an associated reduced intraoperative blood loss of nearly 45% compared with those in NTXA group (8 vs37, 619 ml vs 1125 ml, *P* < 0.05). There were no significant difference in total irrigation fluid between two groups (540 vs 550, *p* >0.05). Additional, patients in NTXA group showed significant decrease of Hb compared with patients in TXA group (5.2 g/dL vs 3.3 g/dL, *P* < 0.05), No significant difference were found in hospitalization time between two groups (6.3vs7.2 days, *P* > 0.05). No minor adverse effects associated with use of TXA were noted.

**Conclusions:**

Use of large dose tranexamic acid routinely seems to be effective and safe in reducing allogenic blood transfusion and blood loss in adolescent idiopathic scoliosis surgery.

## Background

Tranexamic acid (TXA) is a kind of synthetic antifibrinolytic which can inhibit both fibrinolysis and the activation of plasminogen by plasminogen activator to delay fibrinolysis and stop bleeding [1–3]. Its effectiveness in reducing postoperative blood loss and minimizing transfusions has been documented in various surgical subspecialties including cardiac, transplant, and orthopaedic surgery [4, 5]. The effectiveness of antifibrinolytic drugs has been demonstrated in reducing blood loss and the amount of blood transfused in children undergoing scoliosis surgery [6–8], and some current studies also showed efficacy of TXA in reducing allogeneic blood products transfusion in AIS surgery [8–10]. We tried to provide evidence in the form of a historical control study to access efficacy and safety of large dose tranexamic acid in reducing allogeneic blood products in adolescent idiopathic scoliosis surgery.

## Methods

From August 2011 to March 2015, 137 consecutive patients diagnosed with AIS underwent surgery were retrospectively reviewed. Since July 2013, all patients with AIS had received large dose TXA during surgery at our institution. The 71 patients who received intraoperative TXA were categorized as the TXA group. The historical control group (no TXA group) consisted of the remainning 66 AIS patients who underwent surgery without being given TXA. All patients were screened for renal insufficiency and coagulopathy before surgery. Correction of AIS were performed by posterior full pedicle system using the same surgical technique under monitoring of combined MEPs and SEPs, and all surgeries were performed by the same senior doctor. Cell salvage autologous blood recovery system was used in all cases. No preoperative blood donation was obtained from all patients. Outcome measures including preoperative demographics (age, sex, BMI, ASA physical status, properative values of haemoglobin and haematocrit), radiographic parameters (preoperative Cobb angle, postoperative Cobb angle, coronal balance, correction rate), operative data (operative time, mean BP, number of levels fused, screw density), estimated blood loss(EBL), total irrigation fluid, number of patients requiring blood transfusion, drop of Hb (Pre-op Hb-Post-op Hb), mean volume of blood transfusion, hospitalization time, and adverse effects were recorded and compared. The standard TXA dose used in our study was 100 mg/kg intravenously over 15 min after induction of anesthesia and before incision followed by an infusion of 10 mg/kg per hour during surgery until skin closure. Indications for blood transfusion during operation including significant drop in systolic blood pressure (<50 mmHg), any perceived rapid loss of blood, decreased urine output, and alterations in the spinal cord monitoring responses which were decided by the surgeon and anesthetist. After surgery, patients in both groups were managed in the same manner. No suction drain was used in all the patients. Haemoglobin (Hb)/haematocrit(Hct) were routinely checked 1 day after operation. Patients with haemoglobin levels <7 g/dL were considered for transfusion, and patients with haemoglobin levels >7 g/dL showed clinical symptoms including dizziness, excessive fatigue, and hypotension in the case of rule out other posssible causes were also considered as indications for transfusion. No changes were made in the indications for transfusion and hemostatic techniques over the study period. The study has been approved by the Institutional Ethics Committee and informed written consent was obtained from all participants.

Screw density was defined as the total number of screws inserted divided by the total number of level fused.

The coronal spinal balance in the frontal plane was determined on standing radiograph by measuring the C7 plumb line distance from the center sacral line.

Estimated blood volume was calculated using a formula described by Nadler et al. [11]. EBL was calculated by adding the blood volume collected by both the suction and cell-saver (CS) system and by weighing the surgical sponges.

The total irrigation fluid was measured strictly by the nurses prior to use in the operative field.

### Statistical analysis

Descriptive statistics were analyzed by SPSS17.0. Student’s *t*-test was used to compare continuous variables, and a two-sample z test was used to compare sample proportions. Results were considered statistically significant with a p value less than 0.05.

## Results

All the 137 AIS patients were successfully reviewed. There were 71 patients in the TXA group and 66 patients in the NTXA group. All patients showed normal blood urea nitrogen, blood creatinine level, bleeding time, prothrombin time, activated partial thrombin time, international normalized ratio, and serum platelet concentration preoperative. No patient sustained any major intra- or postoperative complications in either group. All patients received general anesthesia with a goal mean arterial pressure of 60 to 70 mmHG and epidural catheters for postoperative pain management. There were no significant differences in the demographics of patients in the two groups (age, sex, BMI, ASA physical status, preoperative values of Hb/Hct, preoperative Cobb angle, postoperative Cobb angle, correction rate, coronal balance, number of levels fused, screw density) (Table 1). The mean surgical time was 209 min in the TXA group and 215 min in the NTXA group (*P >0.05*). Patients in the TXA group showed a significant decrease in transfusion requirements with an associated reduced intraoperative blood loss of nearly 45%compared with those in NTXA group (8vs37, 619 ml vs1125 ml, *P* < 0.05). There were no significant difference in total irrigation fluid between two groups (540 ml vs 550 ml, *p* >0.05). Additional, patients need blood transfusion in NTXA group showed significant decrease of Hb compared with those in tranexamic acid group (5.2 g/dL vs 3.3 g/dL, *P* < 0.05).No significant difference were found in hospitalization time between two groups (6.3 vs 7.2 days, *P* > 0.05) (Table 2). A total of 45 patients need blood transfusion, 8 in the TXA group and 37 in the NTXA group, no statistically significant differences were showed between the recorded parameters of these patiens (*P* > 0.05, Table 3). No minor adverse effects, such as headache, nausea, vomiting, or diarrhea, associated with the use of TXA were noted.

**Table 1:** Mean demographics of patients in the two groups

Data	TXA group (*n* = 71)	NTXA group (*n* = 66)	*P* value
Age (ys)	15.5	16.2	>0.05
Sex (F/M)	(49/22)	(45/21)	>0.05
BMI	17.1	16.9	>0.05
ASA	I	I	>0.05
Pre-op Cobb angle(°)	65.5	62.4	>0.05
Post-op Cobb angle(°)	17.6	18.1	>0.05
Magnitude of correction (%)	72.8	71.6	>0.05
Number of levels fused	13.1	12.8	>0.05
Pedicle screw density (%)	81	79.7	>0.05
Pre-op coronal balance(mm)	12.3	12.9	>0.05
Post-op coronal balance(mm)	4.6	4.8	>0.05
Pre-op Hb (g/dL)	13.2	13.4	>0.05
Pre-op Hct (%)	43.6	42.9	>0.05

**Table 2:** Measured outcomes of patients in the two groups

Data	TXA group (*n* = 71)	NTXA group (*n* = 66)	*P* value
Mean BP (mm Hg)	66.4	68.2	>0.05
Blood transfusion (number)	8	37	<0.05
Estimated blood loss (mL)	619	1125	<0.05
Hb drop (g/dL)	3.3	5.2	<0.05
Operative time (min)	209	215	>0.05
Hospitalization time (days)	6.3	7.2	>0.05
The total irrigation fluid	540	550	>0.05
Post-op Hct (%)	33.1 %	27.8 %	<0.05

**Table 3:** Measured outcomes of blood transfusion patients in the two groups

Data	TXA group (*n* = 8)	NTXA group (*n* = 37)	*P* value
Mean BP (mm Hg)	66.7	67.2	>0.05
Blood transfusion (ml)	373	384	>0.05
Estimated blood loss (mL)	1425	1470	>0.05
Hb drop (g/dL)	6.9	7.3	>0.05
Operative time (min)	215	213	>0.05
Hospitalization time (days)	7.4	7.9	>0.05

## Discussion

Tranexamic Acid (TXA) is a synthetic lysine-analogue antifibrinolytic that was first patented in 1957 [12]. The mechanism of action is the competitive blockade of the lysine-binding sites of plasminogen, plasmin, and tissue plasminogen activator [13]. Use of TXA can reduce blood loss in cardiac surgery, trauma, liver surgery and solid organ transplantation and non-surgical diseases were demonstrated [14, 15]. In a recent meta-analysis of over 100 RCTs that compared TXA vs no TXA or a placebo in more than 10,000 patients undergoing surgery showed overwhelming evidence that TXA reduces the probability of transfusion by 38 % [16]. However, There are few RCTs on TXA use in pediatric scoliosis surgery. The safety and efficacy of TXA for spinal scoliosis surgery are still controversial.

Faraoni et al. [17] concluded that in pediatric spine surgery (mainly scoliosis correction), TXA did decrease blood loss and transfusion requirements. Yang et al. [18] conducted a meta-analysis of intravenous TXA use (*n* = 581) in spinal surgery compared to pediatric scoliosis surgery showed a reduction in postoperative blood loss by 389.21 mL and the amount of blood transfused by 134.55 mL with TXA. Elwatidy et al. [19] reported a significant reduction in EBL in patients who were administered TXA during spinal surgery, whereas Baldus et al. [20] reported no advantage of TXA administration for blood loss during spinal surgery. Dhawale et al. [21] determined that aminocaproic acid resulted in similar EBL to patients who had no AF in a population of pediatric patients with scoliosis secondary to cerebral palsy. A total of 137 AIS patients were retrospectively enrolled in our study, of which 45 patients need blood transfusion, 8 in the TXA group and 37 in the NTXA group. Patients in the TXA group showed a significant decrease in transfusion requirements with an associated reduced intraoperative blood loss of nearly 45%compared with those in NTXA group (8vs37, 619 ml vs1125 ml, *P* < 0.05). Additional, patients need blood transfusion in NTXA group showed significant decrease of Hb/Hct compared with those in tranexamic acid group (5.2 g/dL vs 3.3 g/dL, *P* < 0.05).No significant difference were found in hospitalization time between two groups (6.3 vs 7.2 days, *P* > 0.05) (Table 2).

A wide range of TXA dosing has been advocated, depending on the indication. Most studies use intravenous TXA. The bolus and infusion dosing vary, but pharmacokinetic evidence would suggest the use of a 10 to 15 mg/kg loading dose, followed by an infusion dose of 1 mg/kg/h or repeated bolus dosing [13, 22]. It is now clear from current literature that moderate to high doses of TXA in cardiac surgery are associated with an increased risk of seizures [23, 24]. There is currently no clinical evidence that the use of TXA increases the risk of thromboembolic events, namely myocardial infarction, stroke, deep vein thrombosis or pulmonary embolism according to meta-analyses and clinical trials cited in the trauma and orthopedic [2, 25, 26] settings. In our study, TXA dose 100 mg/kg intravenously over 15 min after induction of anesthesia and before incision followed by an infusion of 10 mg/kg per hour during surgery until skin closure was used as a standard protocol in the TXA group. No minor adverse effects, such as headache, nausea, vomiting, or diarrhea, associated with the use of TXA were noted.

A recent retrospective study of 36,901 patients undergoing elective spinal surgery found that those who received transfusions, even a single unit, had increased length of stay and postoperative morbidity [27]. Dhawale et al. [21] determined that TXA was more effective in reducing blood loss in cerebral palsy patients who underwent scoliosis correction than AFs and EACA, and no significant difference were found between the TXA group and no TXA group in length of hospital stay. In our study, postoperative length of stay was decreased in the group who received TXA, but no statistically significant difference were found between two groups which might due to the sample size and other procedures performed postoperatively. However, it is likely that the reduction in transfusion rates trend to reducing hospitalization time and morbidities with associated improvements in patient outcomes.

Ialenti et al. [28] reported that prolonged operative time is associated with increased blood loss. Mark J et al. [29] reported that The use of tranexamic acid has also led to a statistically significant reduction in operation time (188 vs 223 min). Frederic et al. [30] reported that the length of surgery, in minutes, was not significantly different between the non-TXA and TXA patients. There were no significant difference between two groups in operative time in this study. We think a bipolar sealer played an important role in assisting hemostasis which might result in shorten operative time, and this is familiar to the former study performed by Mankin [31].

The limitations of this study are inherent to its retrospective designed with historical control group. Another potential limitation was the indications for transfusion were not defined with objective parameters.

## Conclusion

Our retrospective study designed with historical control group showed that large dose TXA seems to be effective and safe in reducing allogenic blood transfusion and blood loss in adolescent idiopathic scoliosis surgery. Prophylactic TXA may provide a worthwhile reduction of excessive blood loss and decrease the need for blood transfusion during idiopathic scoliosis surgery in adolescent patients.

### Ethics approval and consent to participate

The study was approved by the medical ethics committee of the first affiliated hospital of Sun Yat-Sen University.

### Data availability statement

We had full access to all of the data in the study and take responsibility for the integrity of the data and the accuracy of the data analysis. The data is available on request from the corresponding author.

## Abbreviations

AIS: adolescent idiopathic scoliosis
EBL: estimated blood loss
NTXA: no tranexamic acid
TXA: tranexamic acid
